# With Risk of Reinfection, Is COVID-19 Here to Stay?

**DOI:** 10.1017/dmp.2020.274

**Published:** 2020-07-27

**Authors:** Parnian Jabbari, Nima Rezaei

**Affiliations:** Research Center for Immunodeficiencies, Children’s Medical Center, Tehran University of Medical Sciences, Tehran, Iran; Network of Immunity in Infection, Malignancy and Autoimmunity (NIIMA), Universal Scientific Education and Research Network (USERN), Tehran, Iran; Department of Immunology, School of Medicine, Tehran University of Medical Sciences, Tehran, Iran

**Keywords:** COVID-19, reinfection, latent infection, pandemics, SARS-CoV-2

Many factors have served the swift transformation of the coronavirus disease (COVID-19) from an endemic to a pandemic.^1^ Some can be attributed to characteristics of the virus, severe acute respiratory syndrome coronavirus 2 (SARS-CoV-2), such as high basic reproducing number (R_0_) of about 2 to 6 and transmissibility despite absence of clinical manifestations.^2^ However, our assumptions about the pandemic, most of which are falsified or questioned, at this point, also played a major role. One such assumption was that SARS-CoV-2, similar to H1N1 virus, will attenuate in high temperatures, while we are now observing the second wave of the pandemic in some countries, despite rising temperatures. Another assumption was reaching herd immunity for SARS-CoV-2 as the number of the affected individuals would rise, thus restrict the transmission of virus due to the increased number of immune hosts and decreased number of susceptible ones.^2^ However, a key concept in establishing and maintaining herd immunity is a strong immunization against the pathogens which lasts long enough so that the fraction of the population immune to the virus surpasses the herd immunity threshold. Therefore, persistence of immunoglobulin concentrations is one of the factors that determines the possibility of reaching herd immunity or periodic outbreaks.^2^

In the majority of individuals infected with SARS-CoV-2, neutralizing immunoglobulin (IgM and IgG) levels rise within days to weeks of symptom onset.^3^ These antibodies have reported to produce immunity to reinfection in primates re-challenged with SARS-CoV-2 at 28 days after the initial infection.^4^ However, unlike many other respiratory viruses resulting in immunoglobulin concentrations that last for several months, neutralizing immunoglobulins against SARS-CoV-2 persist for about 40 days.^4^ On the other hand, positive RNA tests, despite seropositivity for IgG after primary infection, have been reported.^5^ Even though such cases have been interpreted as silent carriers or low reliability for the commercially available kits and sampling errors, the time window between the primary infection and the second positive RNA test, which is about 2 months, in some cases, can be suggestive of reinfection with the virus, or reactivity of a latent infection with SARS-CoV-2.

Even though studies are promising for an effective vaccine in the next 12 to 18 months,^2^ the presence of more than 80 genotypical variants of the virus, possibility of reinfection, and short duration of seropositivity for neutralizing antibodies raise the concern that vaccination may not result in an effective and long-term immunity against SARS-CoV-2. Furthermore, immunoglobulin levels may not correlate with viral shedding and risk of transmissibility of SARS-CoV-2.^5^ Also, the short duration of immunity against the virus may not allow for increasing homogeneity of affected populations in a specific time frame. Therefore, herd immunity may not be achieved as reinfection may occur even in the presence of neutralizing antibodies. These factors raise concerns that eliminating the COVID-19 pandemic may not be as feasible as once assumed and that we must rely more on prevention of transmission until more aspects of the virus and its pathogenicity are discovered.

## References

[1] Hanaei S , Rezaei N. COVID-19: developing from an outbreak to a pandemic. Arch Med Res. 2020;epub. doi: 10.1016/j.arcmed.2020.04.021. PMC7219390 32405122

[2] Randolph HE , Barriero LB. Herd immunity: understanding COVID-19. Immunity. 2020;52(5):737-741. 32433946 10.1016/j.immuni.2020.04.012 PMC7236739

[3] Lotfi M , Rezaei N. SARS-CoV-2: a comprehensive review from pathogenicity of the virus to clinical consequences. J Med Virol. 2020;epub. 10.1002/jmv.26123. PMC7300719 32492197

[4] Kirkcaldy RD , King BA , Brooks JT. COVID-19 and postinfection immunity: limited evidence, many remaining questions. JAMA. 2020;epub. doi: 10.1001/jama.2020.7869. PMC8596300 32391855

[5] Roy S. COVID-19 reinfection: myth or truth? SN Compr Clin Med. 2020;2:710-713. 10.1007/s42399-020-00335-8 PMC7255905 32838134

